# Multiple-Strain Colonization in Nasal Carriers of Staphylococcus aureus

**DOI:** 10.1128/JCM.03254-13

**Published:** 2014-04

**Authors:** A. A. Votintseva, R. R. Miller, R. Fung, K. Knox, H. Godwin, T. E. A. Peto, D. W. Crook, R. Bowden, A. S. Walker

**Affiliations:** a Nuffield Department of Clinical Medicine, University of Oxford, John Radcliffe Hospital, Oxford, United Kingdom; b National Institute for Health Research (NIHR) Oxford Biomedical Research Centre, John Radcliffe Hospital, Oxford, United Kingdom; c Wellcome Trust Centre for Human Genetics, University of Oxford, Oxford, United Kingdom; d Nuffield Department of Primary Care Health Sciences, University of Oxford, Oxford, United Kingdom

## Abstract

Staphylococcus aureus is a commensal that can also cause invasive infection. Reports suggest that nasal cocolonization occurs rarely, but the resources required to sequence multiple colonies have precluded its large-scale investigation. A staged protocol was developed to maximize detection of mixed-*spa*-type colonization while minimizing laboratory resources using 3,197 S. aureus-positive samples from a longitudinal study of healthy individuals in Oxfordshire, United Kingdom. Initial typing of pooled material from each sample identified a single unambiguous strain in 89.6% of samples. Twelve single-colony isolates were typed from samples producing ambiguous initial results. All samples could be resolved into one or more *spa* types using the protocol. Cocolonization point prevalence was 3.4 to 5.8% over 24 months of follow-up in 360 recruitment-positives. However, 18% were cocolonized at least once, most only transiently. Cocolonizing *spa* types were completely unrelated in 56% of samples. Of 272 recruitment-positives returning ≥12 swabs, 166 (61%) carried S. aureus continuously but only 106 (39%) carried the same single *spa* type without any cocolonization; 31 (11%) switched *spa* type and 29 (11%) had transient cocarriage. S. aureus colonization is dynamic even in long-term carriers. New unrelated cocolonizing strains could increase invasive disease risk, and ongoing within-host evolution could increase invasive potential, possibilities that future studies should explore.

## INTRODUCTION

Staphylococcus aureus is a commensal organism that is also responsible for a wide range of human infections, including serious invasive disease. Although other body sites may also be clinically important (1–3), nasopharyngeal carriage is a key source of clinical S. aureus infection, both for the carrier, since >80% of clinical infections are with an endogenous strain, and for other individuals, since recent acquisition increases the risk of clinical infection (4–7).

Some bacteria (e.g., coagulase-negative staphylococci) sustain predominantly polyclonal nasal colonization (8, 9), but historically, each S. aureus carrier has been assumed to be colonized by a single strain. The near-universal adoption of S. aureus screening protocols that type only 1 to 3 colonies per sample (4, 5, 10–13) has, perhaps unsurprisingly, supported this view, although there appears to be little direct evidence in the literature. Multiple colonization could reduce the power of studies relying on typing to infer transmission (since minority strains might also be transmitted) and could limit our understanding of the interactions between colonizing organisms, including carriage dynamics, evolution, and horizontal genetic transfer through homologous recombination (14, 15).

Despite extensive investigation of S. aureus carriage in hospitals and communities, few studies have characterized the prevalence and properties of multiple-strain colonization. Among these are descriptions of carriage of more than one strain based on single colonies isolated at different time points (13, 16) and of simultaneous colonization of different body sites with different strains (17–19), including a report of cocolonization in 38 (30%) children in a cross-sectional study of 125 nasal and perianal samples (20). The S. aureus nasal cocolonization rate appears to have been estimated rarely; in one cross-sectional study, >1 strain was detected in 9% (14/148) of carriers based on pulsed-field gel electrophoresis of three colonies (7 [5%] differing by >3 bands and 4 [3%] also differing by *spa*, *agr*, and multilocus sequence typing [MLST]) (21). In another, where the number of colonies typed at baseline visit was reported as “at least 2” (22) and “8” (23), only 2/89 (2%) participants had >1 strain.

Here, we use *spa* typing to systematically investigate multiple-strain colonization at the same time point, and over time, in a large longitudinal cohort of healthy nasal S. aureus carriers in Oxfordshire, United Kingdom (24). Our objectives were (i) to develop an efficient protocol to detect mixed-*spa*-type colonization consistently, (ii) to estimate the prevalence of multiple-strain community-associated carriage, and (iii) to characterize individual cases of multiple colonization, in order to better understand the nature and dynamics of mixed S. aureus communities.

## MATERIALS AND METHODS

Nasal swabs were collected from individuals recruited from five Oxfordshire general practices (GPs [family doctors]) in the Thames Valley Primary Care Research Partnership between December 2008 and December 2009. Eligible participants were adults aged ≥16 years. Two hundred participants were recruited from each GP practice, in age/sex strata approximately representing the United Kingdom population. To increase numbers of younger participants, students registering at one practice were recruited during the University Freshers' week (24). On recruitment, a cotton swab was inserted into one and then the other of the participant's nostrils and rotated three times under the supervision of a research nurse. Participants were trained in self-swabbing, and all of those positive for S. aureus at recruitment (*n* = 360) (recruitment-positives) plus additional recruitment S. aureus negatives from the last general practice (*n* = 211) (recruitment-negatives) were sent a self-swabbing kit after 1 month and then every 2 months for 2 years, with a subset followed thereafter. This analysis included isolates from the first 24 months of follow-up. Swabs were returned by post in charcoal medium (typically <1 week) and stored on receipt at 4°C before processing (<1 week).

### Bacterial isolates.

As the study objective was to investigate S. aureus dynamics, isolation protocols focused on identifying all strains, even those present at low frequencies. To increase sensitivity, each nasal swab was placed in 5% NaCl enrichment broth (E&O Laboratories) and incubated overnight at 37°C. A loopful of broth was subcultured onto Sa*Select* chromogenic agar (Bio-Rad) and incubated at 37°C overnight. Pink colonies regarded as S. aureus were positively identified using a Prolex Staph Xtra Latex kit (Pro-Lab Diagnostics) and catalase, DNase, and tube coagulase tests. Methicillin resistance was tested on Columbia agar with 5.0% salt (Oxoid) with 1 μg oxacillin discs (BD). S. aureus organisms isolated from swabs returned before 1 July 2009 were stored (and typed) as single colonies, with multiple colonies typed only if they demonstrated different morphology. Subsequently, mixed glycerol stocks of S. aureus cultures were prepared by suspending several loopfuls of bacteria taken by sweeping across the Sa*Select* plate in 1.5 ml of saline with 200 μl of 45% glycerol for storage at −80°C. Taking a sweep across the plate rather than picking a single colony for glycerol stocks allowed us to maintain the genetic diversity of nasal strains in the sample for later analyses. Crude S. aureus DNA extracts (“boilates”) were made from single bacterial colonies before 1 July 2009 (chosen to represent the diversity of color and size on the plate) or subsequently from mixed glycerol stocks revived on Sa*Select* plates (see Results for details of the standardized protocol). With a 1-mm loop, a small amount of bacteria was emulsified into 60 μl of Tris-EDTA (TE) buffer (Sigma-Aldrich) and then heated in a thermocycler at 99.9°C for 10 min and centrifuged at maximum speed for 2 min. Fifty microliters of supernatant was removed without disturbing the pellet and stored at −20°C for use as a PCR template.

### *spa* typing.

Given the study size, *spa* typing, a highly discriminatory single-locus typing approach targeting sequence variation in the polymorphic Xr region of the staphylococcal protein A (*spa*) gene (25, 26), was used to define discrete strain types. The *spa* region was amplified with primers 1095F, 5′-AGACGATCCTTCGGTGAGC-3′, and 1517R, 5′-GCTTTTGCAATGTCATTTACTG-3′ (25, 27). PCR mixtures consisted of 0.25 mM deoxynucleoside triphosphates (dNTPs), 0.5 U of GoTaq Flexi DNA polymerase (Promega), GoTaq Flexi Buffer, 2.5 mM MgCl_2_, and 0.25 μM primers in a volume of 10 μl. PCR conditions were 94°C for 2 min; 35 cycles each of 94°C for 30 s, 50°C for 30 s, and 72°C for 60 s; and a final extension at 72°C for 5 min. PCR products were purified using AMPure XP beads (Beckman Coulter).

Samples were sequenced with the PCR primers: in most cases, only the 1095F primer was required to determine the *spa* type. Sequencing reactions used BigDye v3.1 (Applied Biosystems), and reaction mixtures were cycled using 30 cycles of 96°C for 10 s, 50°C for 5 s, and 60°C for 2 min. Products were purified with CleanSEQ beads (Beckman Coulter) and sequenced on an ABI 3730 DNA analyzer (Applied Biosystems). For isolates that did not yield clean sequence with the original primers, an alternate forward primer, spaT3-F (5′-CAACGCAATGGTTTCATCCA-3′), binding to a sequence encoding part of 5 IgG-binding domains, was used with 1517R in PCR (A. A. Votintseva, R. Fung, R. R. Miller, K. Knox, H. Godwin, D. H. Wyllie, R. Bowden, D. W. Crook, and A. S. Walker, submitted for publication). Only the 1517R primer was then used to determine the sequence.

Chromatograms were analyzed using Ridom StaphType v2.0.3 software (Ridom GmbH). The relationships between *spa* types were investigated using the BURP clustering algorithm (28).

### Statistical analyses.

Statistical analyses used Stata 11.2. Proportions were compared between recruitment-positives and recruitment-negatives using chi-square tests (unless any cell frequency was <5 or any cell percentage was <5% when Fisher's exact tests were used), and continuous variables using rank sum tests. Generalized estimating equations (GEE) with independent working correlation structure were used to investigate whether the proportion of participants with mixed carriage varied over time in the study.

## RESULTS

### Protocol for resolving multiple-strain colonization.

**FIG 1:**
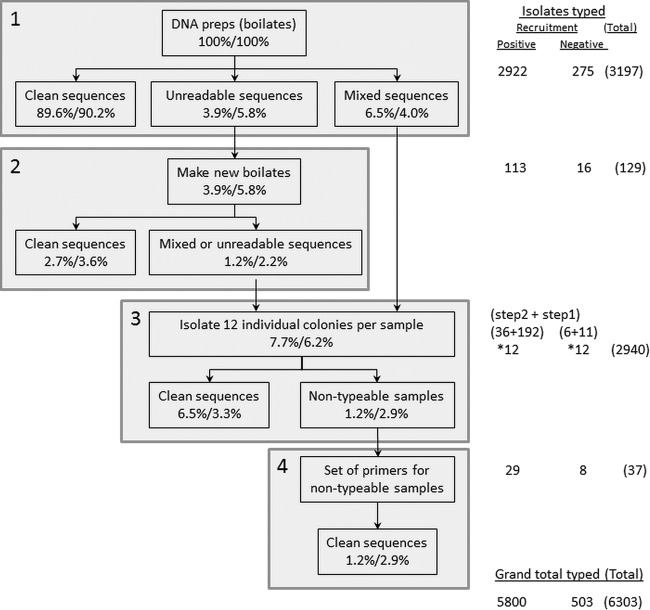
Enhanced *spa* typing protocol. Up to four attempts (steps 1 to 4) were made to *spa* type each sample. (Step 1) For every sample, a single DNA boilate derived from pooled glycerol stock (see Materials and Methods) was *spa* typed, with three possible outcomes (see Fig. S1 in the supplemental material): (i) “clean,” unambiguous low-background sequence traces with successful assignment of a *spa* type were interpreted as a lack of evidence for mixed infection; (ii) “mixed” sequence traces with two or more superimposed peaks for at least some of the trace were interpreted as mixed infections and reserved for deeper sequencing in step 3; (iii) unreadable, low-quality traces were usually caused by failed DNA isolation and so were repeated in step 2. (Step 2) Repeat typing was attempted on new pooled DNA boilates, resulting in ∼80% of cases failing step 1 being successfully assigned a *spa* type. (Step 3) Twelve individual colonies were prepared and typed for each of the samples that yielded mixed traces in step 1 or mixed or unreadable traces in step 2. (Step 4) Individual-colony samples that were nontypeable in step 3 were retyped using a novel upstream forward primer (Votintseva et al., submitted). Note that numbers (%) refer to isolates from participants who were S. aureus positive or negative, respectively, at recruitment. Percentages are of all samples typed. “*12” indicates that 12 individual colonies were isolated and *spa* typed for each sample.

Conventionally, large-scale *spa*-typing studies have analyzed 1 to 3 single-colony isolates for each S. aureus-positive sample (4, 5, 10–13). Sequencing many colonies individually for every sample in a large study would be unnecessary if only a small fraction contained multiple strains, as well as being expensive and time-consuming. However, if only a small fraction contained multiple strains, then sequencing a sweep across a culture plate would mostly sample only a single strain, providing the “single”-colonization *spa* type but also allowing samples with mixed colonization to be identified by their unreadable/mixed sequence traces. Therefore, we developed a staged protocol to maximize detection of mixed colonization while minimizing laboratory resources (Fig. 1). This used pooled material from each sample for initial typing, identifying a single unambiguous strain in most samples. Samples producing ambiguous initial typing results were resolved by sequencing several (typically 12) single-colony isolates.

Over the first 24 months, 3,303 and 275 S. aureus-positive swabs were returned from individuals who were S. aureus positive or negative on recruitment, respectively. The 381 samples that had been collected before 1 July 2009 from recruitment-positives were initially analyzed and stored only as single-colony isolates and so could not be investigated for mixed infection using the staged protocol. DNA from pooled colonies generated from the other 3,197 samples collected after 1 July 2009 and stored as mixed glycerol stocks was analyzed according to the staged protocol (Fig. 1). Of samples from recruitment-positives, 89.6% yielded an unambiguous *spa* type (step 1, “clean sequences”), suggesting nil to low levels of minority cocolonizing strains (the percentage was similar in recruitment-negatives [chi-square, *P* = 0.75 versus recruitment-positives]). (Examples of *spa*-type traces are given in Fig. S1 in the supplemental material.) Of samples from recruitment-positives, 3.9% initially gave unreadable sequences from a plate sweep but 2.7% provided clean sequences from a second sweep (step 2). Of samples from recruitment-positives,7.7% could not be typed from these two initial rounds; 6.5% could be resolved into *spa* types by typing 12 colonies individually (step 3, “clean sequences”). The choice to sequence 12 colonies from samples with mixed/repeatedly unreadable sequences was partly made from convenience (since it represents one row in a plate, thus reducing laboratory error) and partly from the theoretical probability of identifying a minor cocolonizing *spa* type given standard sampling variability (see Fig. S2 in the supplemental material). The remaining 1.2% of samples from recruitment-positives would formerly have been designated nontypeable and required a novel forward primer, spaT3-F (5′-CAACGCAATGGTTTCATCCA-3′), to identify *spa* types (step 4, “clean sequences”) (Votintseva et al., submitted). The protocol resolved 100% of samples from both recruitment-positives and recruitment-negatives into one or more *spa* types, with similar performances in the two groups. The total culturing/typing effort was 6,303 isolates from the original 3,197 S. aureus-positive samples, a 2-fold increase (Fig. 1), with most additional effort expended typing 12 individual colonies from isolates with mixed or repeatedly unreadable sequences.

Of the 208 samples where multiple individual colonies were successfully typed in step 3 (Fig. 1), the same *spa* type was identified in all colonies in 69 (33%) (34% recruitment-positives versus 22% recruitment-negatives, exact *P* = 0.72). These may have represented very-low-level cocolonization, in which the multiple *spa*-typed colonies had by chance not included the minority strain(s), since the isolates had failed mixed-colony sequencing with clear double peaks once or twice, which was unlikely by chance. The proportion of typed isolates which made up the majority population varied substantially (see Fig. S3 in the supplemental material); excluding samples where only one *spa* type was recovered from multiple picks, the median frequency of the dominant *spa* type was 0.75 (interquartile range [IQR], 0.60 to 0.83; range, 0.42 to 0.96) (rank sum, *P* = 0.43, comparing recruitment-positives with recruitment-negatives). A maximum of four different *spa* types were retrieved from multiple picks from a single sample.

To confirm that sequencing a pooled extract was not missing diversity in a substantial proportion of samples, we sequenced 12 colonies from a random selection of 32 samples appearing to contain a single strain (yielding clean sequence traces in step 1, Fig. 1). No extra *spa* types were observed among the 384 sequences (each sample produced 12 colonies with identical sequences), providing an upper 97.5% confidence limit of 11% around the observed 0% with initially clean sequences in fact having mixed-strain carriage.

### Mixed-strain colonization according to *spa* type.

During the first 24 months in the study, 4,257 and 2,352 samples were submitted from 360 S. aureus recruitment-positives and 211 recruitment-negatives, respectively (median, 14 [IQR, 11 to 14] swabs per individual). Of the samples, 3,303 (77.6%) and 275 (11.7%), respectively, were S. aureus positive and *spa* typed as described above. Among 161 samples with multiple strains identified (including from typing strains with differing morphology before 1 July 2009), 2 *spa* types were detected in 142 (88%) samples, 3 *spa* types were detected in 17 (10%) samples, and 4 *spa* types were detected in two samples (1%).

**FIG 2:**
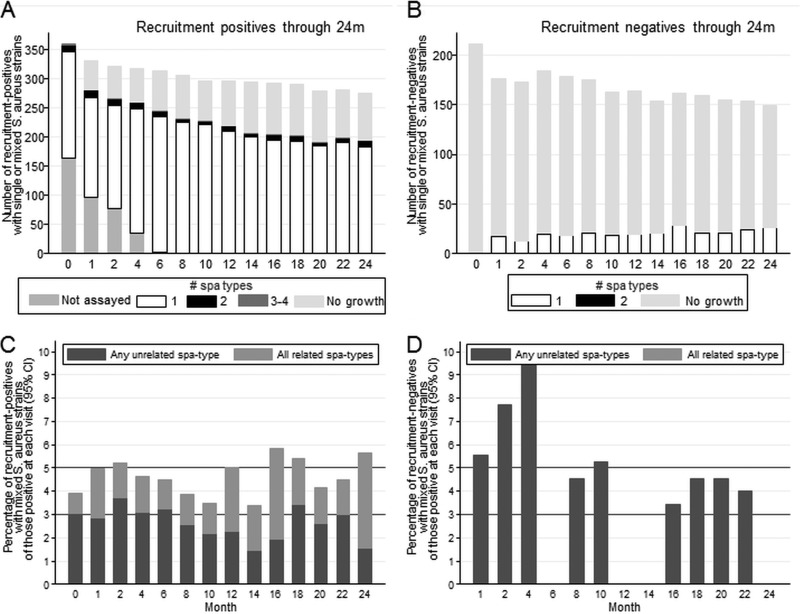
Mixed-strain colonization over follow-up in participants positive and negative for S. aureus at recruitment. (A) Number of recruitment-positives with single- or mixed-strain colonization over time. (B) Number of recruitment-negatives with single- or mixed-strain colonization over time. (C) Percentage of recruitment-positives with S. aureus isolated at each time point who had mixed-strain colonization with any unrelated strains versus all related strains over time. Lines at 3 and 5% are for reference. (D) Percentage of recruitment-negatives with S. aureus isolated at each time point who had mixed-strain colonization with any unrelated strains versus all related strains over time. Lines at 3 and 5% are for reference. Relatedness was predefined as a BURP distance of ≤6.

Of samples from recruitment-positives and recruitment-negatives, 7.7% and 6.2%, respectively, had some evidence of mixed colonization (step 3, Fig. 1); multiple *spa* types were actually detected in 4.6% of samples from recruitment-positives versus 3.6% from recruitment-negatives (*P* = 0.52). The prevalence of mixed-strain colonization was approximately constant across follow-up (Fig. 2; GEE, *P* > 0.9, testing for variation over time), ranging from 3.4 to 5.8% in recruitment-positives (*n* > 190 at each time point) but with point prevalence varying more in the smaller number of recruitment-negatives subsequently observed to carry S. aureus. Mixed carriage was observed less frequently in positive samples following one or two immediately preceding negative samples (odds ratio [OR] versus previous positive sample, 0.60 [95% confidence interval {95% CI}, 0.29 to 1.24] and 0.32 [95% CI, 0.08 to 1.33], respectively), but differences did not reach statistical significance (*P* = 0.16 and *P* = 0.12, respectively).

However, compared to the point prevalence of 3 to 6%, a substantially greater proportion of participants had mixed colonization at any follow-up, 66 (18.3%) recruitment-positives versus 8 (11.3%) of the 71 recruitment-negatives subsequently being observed to carry S. aureus (chi-square, *P* = 0.15). Most had mixed colonization only transiently, in a median of 11% (IQR, 7 to 23%; range, 7 to 100%) of their S. aureus-positive samples (rank sum, *P* = 0.38). A further 57 (16%) recruitment-positives and 21 (30%) recruitment-negatives had different *spa* types isolated only at different time points but no evidence of mixed colonization at one time point.

### MRSA colonization in healthy individuals.

Methicillin-resistant S. aureus (MRSA) strains were found in 10 (2.8%) recruitment-positives and no recruitment-negatives. Patterns of MRSA colonization (Tables 1) were representative of S. aureus carriage in general. Three participants (A to C) had continuous carriage with a single *spa* type throughout the study, one (B) with mixed colonization with a closely related MRSA strain at the last time point. Two participants (D and E) had continuous S. aureus carriage, but with evidence of mixed colonization followed by strain switching; one switch (D) was between two closely related MRSA strains (differing only by duplication of three repeats), but the other (E) was from a MRSA strain to an unrelated methicillin-sensitive strain. Two (F and G) had continuous MRSA in 7 swabs which was then consistently lost, one (G) with mixed colonization with a closely related MRSA strain at the last positive time point only. Two (H and I) had MRSA strains only transiently at recruitment (one with no growth on any further swabs and the other with no growth from months 1 to 4 and then continuous colonization with a methicillin-sensitive strain). The final patient (J) had MRSA only at month 22, having previously been consistently colonized with methicillin-sensitive S. aureus.

**TABLE 1:** Patterns of carriage in individuals ever colonized by a MRSA strain^*a*^

Carriage pattern	Patient identifier	Type at time point (mo)														Type	Repeats
		0	1	2	4	6	8	10	12	14	16	18	20	22	24		
Continuous carriage with one MRSA *spa* type throughout the study	A	t018	t018	t018	t018	t018	t018	t018	t018	t018	t018	t018	t018	t018	t018	t018	15-12-16-02-16-02-25-17-24-24-24
	B	t379	t379	t379	t379	t379	t379	t379	t379	t379	t379	t379	t379	t379	t379*	t379	26-23-23-13-23-31-29-17-25-17-25-16-28
															t608*	t608	26-23------------31-05-17-25-17-25-16-28
	C	t032	t032	t032	t032	t032	t032	t032	t032	t032	t032	t032	t032	t032*	t032	t032	26-23-23-13-23-31-29-17-31-29-17-25-17-25-16-28
Continuous carriage with mixed colonization and then strain switch (one switch to closely related *spa* type, one switch to an unrelated *spa* type)	D	t032	t032*	t032												t032	26-23-23-13-23-31-29-17-31-29-17-25-17-25-16-28
			t1036*		t1036	t1036	t1036	t1036	t1036	t1036	t1036	t1036	t1036*	t1036	t1036	t1036	26-23-23-13------------17-31-29-17-25-17-25-16-28
	E	t032	t032	t032	t032	t032	t032	t032	t032*							t032	26-23-23-13-23-31-29-17-31-29-17-25-17-25-16-28
									t2271*	t2271	t2271	t2271	t2271	t2271	t2271	t2271	15-12-16-16-02-25-17-24-24
Continuous carriage followed by confirmed loss	F	t032	t032	t032	t032	t032	t032	t032	NG	NG	NG	NG	NG	NG	NG	t032	26-23-23-13-23-31-29-17-31-29-17-25-17-25-16-28
		t012	t012	t012	t012	t012		t012	t012*							t012	15-12-16-02-16-02-25-17-24-24
	G						t748			NG	NG	NG	NG	NG	NG	t748	15-12-------------------17-24----
									t021*							t021	15-12-16-02-16-02-25-17-24----
MRSA transiently at recruitment (one subsequently continuously colonized with related but methicillin-sensitive *spa* type, one not subsequently colonized)	H	t018	NG	NG	NG											t018	15-12-16-02-16-02-25-17-24-24-24
						t012	t012	t012	t012	t012	t012	t012	t012	t012	t012	t012	15-12-16-02-16-02-25-17-24-24----
	I	t032	NG	NG	NG	NG	NG	NG	NG	NG	NG	NG	NG	NG	NG	t032	26-23-23-13-23-31-29-17-31-29-17-25-17-25-16-28
MRSA transiently postrecruitment (initially continuously colonized with methicillin-sensitive strain)	J	t213	t213	t213	t213	t213	t213	t213	t213	t213	t213	t213	NG		NG	t213	07-23-12-21-24-33-22-17
														t032*		t032	26-23-23-13-23-31-29-17-31-29-17-25-17-25-16-28

a Underlining indicates MRSA strains; asterisks indicate strains with mixed/unreadable traces on one or two boilates, from which 12 picks were sequenced. NG, no growth.

### Mixed-strain colonization by related versus unrelated *spa* types.

**FIG 3:**
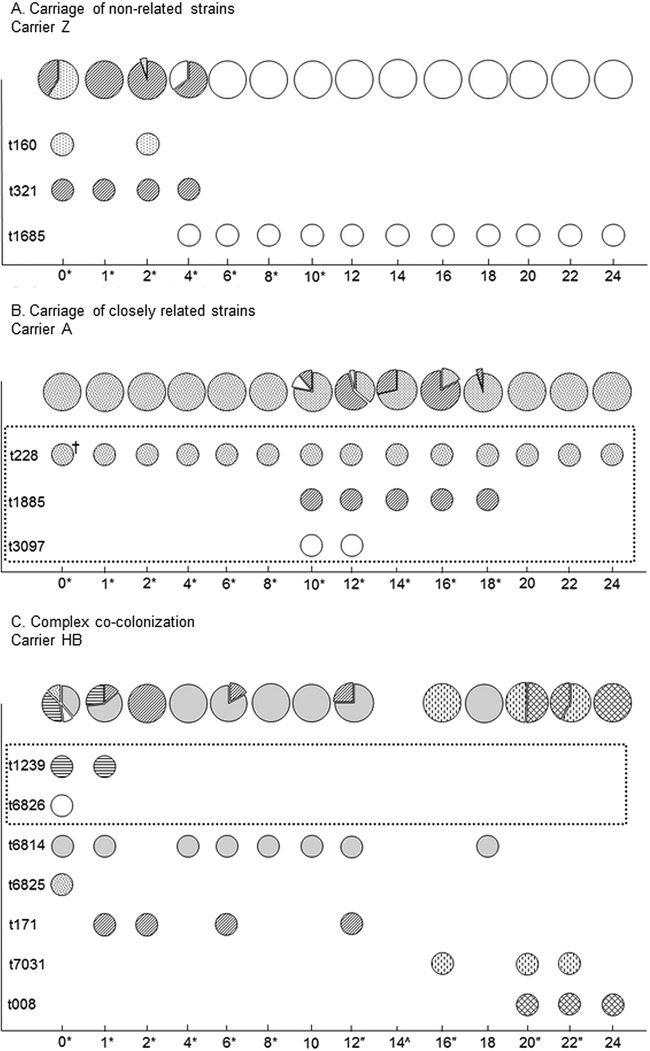
Longitudinal assessment of cocolonization in selected individuals. Related *spa* types are enclosed in a dotted-line box. Asterisks indicate time points with 24 individual colony picks; quotation marks indicate time points with 12 individual colony picks; a caret indicates no growth of S. aureus; a dagger indicates single-colony glycerol stock. Diagrams represent proportions of different *spa* types per time point.

Twenty-four single-colony isolates were *spa* typed from eight recruitment-positives with evidence of mixed-strain colonization in several successive samples to further investigate patterns of S. aureus cocolonization (Fig. 3; see also Fig. S4 in the supplemental material). We used the BURP algorithm (28) with a prespecified maximum penalty of 6 to distinguish multiple separate colonization events (unrelated types) from colonizations in which microevolution could have occurred at the *spa* locus (related types). Half (16) of the 32 identified *spa* types came from 6 groups of related strains, thus representing 22 distinct S. aureus acquisitions. The observed proportions of different *spa* types varied substantially between successive samples. Some *spa* types disappeared for one or two time points and then reappeared, presumably because their frequency fell below a level at which they could be reliably detected among 24 single colonies, although loss and recolonization from another body site or individual cannot be excluded. As with MRSA, cocolonization by related strains was often dominated by a single long-lasting *spa* type, with variants arising at low frequency and disappearing again.

**FIG 4:**
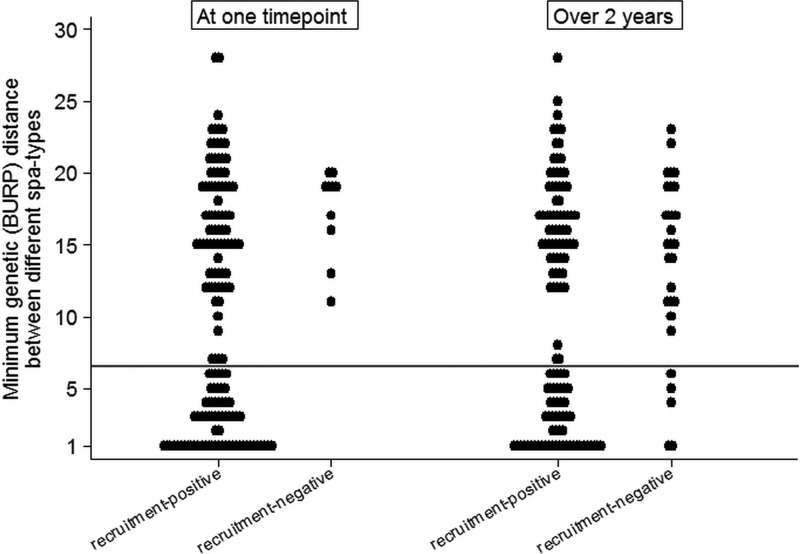
Relatedness of S. aureus strains colonizing a participant at one time point (left) or observed within an individual over 24 months (right). Minimum and maximum distance between *spa* types observed within one individual at one time point (left) or over the study (right). The reference line indicates the predefined relatedness threshold (BURP = 6).

Overall, of the 151 samples where multiple colonizing *spa* types were observed in recruitment-positives, 84 (56%) were only with unrelated types, 62 (41%) were only with related types, and only 5 (3%) were with a mixture of related and unrelated types (Fig. 4, left; see also Table S1a in the supplemental material). In contrast, only unrelated types were observed at all 10 samples where recruitment-negatives had multiple colonizing *spa* types (exact *P* = 0.02). There was no clear pattern of unrelated versus related mixed-strain carriage across samples (Fig. 2C and D). One hundred twenty-three recruitment-positives and 29 recruitment-negatives had >1 *spa* type observed over 24 months, at the same or different time points; 68 (55%) versus 24 (83%), respectively, were only ever colonized with unrelated types, 34 (28%) versus 3 (10%) were only ever colonized with related types, and 21 (17%) versus 2 (7%) were colonized with both related and unrelated types (exact *P* = 0.03; similar results for other penalty thresholds; see Table S1 in the supplemental material).

Considering carriage patterns in the 272 recruitment-positives who returned ≥12 swabs over 24 months, 166 (61%) carried S. aureus continuously. Only 106 (39%) carried the same *spa* type with no other *spa* types observed; 31 (11%) had a switch in *spa* type during continuous carriage, and 29 (11%) maintained the original *spa* type despite transient cocarriage (Table 2). Complete loss and gain of S. aureus occurred with and without cocarriage with related or unrelated *spa* types.

**TABLE 2:** S. aureus carriage patterns in participants returning ≥12 swabs over 24 months^*a*^

Pattern	No. (%)		
	Total (*n* = 331)	Recruitment-positives (*n* = 272)	Recruitment-negatives (*n* = 59)
Continuous carriage			
With 1 *spa* type only (no other *spa* types)	107 (32)	106 (39)	1 (2)
With 1 *spa* type			
And transient related *spa* type(s)	16 (5)	16 (6)	0
And transient unrelated *spa* type(s)	14 (4)	13 (5)	1 (2)
Switch to new related *spa* type (no other *spa* types)	8 (2)	8 (3)	0
Switch to new unrelated *spa* type (no other *spa* types)	24 (7)	23 (8)	1 (2)
Confirmed loss of carriage			
With 1 *spa* type only (no other *spa* types)	46 (14)	46 (17)	0
With 1 *spa* type			
Transient related *spa* type(s)	3 (1)	3 (1)	0
Transient unrelated *spa* type(s)	11 (3)	9 (3)	2 (3)
Confirmed gain of carriage			
With 1 *spa* type only (no other *spa* types)	11 (3)	0	11 (19)
With 1 *spa* type			
Transient related *spa* type(s)	3 (1)	2 (1)	1 (2)
Transient unrelated *spa* type(s)	9 (3)	5 (1)	4 (7)
Confirmed loss and gain (or vice versa)			
Of the same *spa* type			
No other *spa* types	17 (5)	11 (4)	6 (10)
Transient unrelated *spa* type(s)	8 (2)	4 (1)	4 (7)
Confirmed gain and loss of the same *spa* type followed by gain of multiple unrelated *spa* types	3 (1)	0	3 (5)
Two separated carriage episodes			
Related *spa* types	1 (0.3)	0	1 (2)
Unrelated *spa* types	11 (3)	11 (4)	0
Transient but multiple intermittent observations			
Of 1 *spa* type only (no other *spa* types)	5 (2)	2 (1)	3 (5)
Of >1 related *spa* type	9 (3)	3 (1)	6 (10)
Of >1 unrelated *spa* type	4 (1)	2 (1)	2 (3)
Transient carriage at recruitment only	8 (2)	8 (3)	0
Transient carriage once postrecruitment only	13 (4)	0	13 (22)

a Continuous carriage was defined as allowing single intermittent negative swabs. Confirmed loss and *spa* type gain required 2 consecutive observations of no growth or the same *spa* type, respectively.

## DISCUSSION

Historically, S. aureus colonization has been assumed to be with a single strain, an assumption reinforced by the practice of typically typing only 1 to 3 colonies. Sequencing 3 colonies has only an ∼75% chance of identifying two strains present at approximately equal frequencies, dropping to <50% for minor strain frequencies of 0.1 to 0.3 (see Fig. S2 in the supplemental material). Sequencing 12 rather than 3 colonies increases the probability of detecting such mixed-strain colonization by ∼40% but is clearly an infeasible large-scale approach. Here, using only twice the typing effort, we demonstrate conclusively that mixed S. aureus strain colonization can be detected using a standardized protocol in ∼5% of individuals at any time point, regardless of whether they were carrying S. aureus at study recruitment, with nearly 20% of recruitment-positives having evidence of multiple-strain colonization at some point over 24 months. Our estimates of mixed-carriage prevalence are similar to or somewhat higher than (but within the confidence limits of) those of previous studies (21–23) but provide additional confidence from the longitudinal study design. Inspection of individuals' *spa* types, including deeper typing of a subset (Fig. 3), suggested that many probably had true continuous cocolonization rather than separate episodes. As sampling was bimonthly after month 2, it seems likely that even more mixed colonization could have been missed.

Because our protocol focuses on the ∼7% of samples that have some evidence of multiple-strain colonization (Fig. 1), it effectively detects moderate levels of cocolonization and provides a more accurate picture of S. aureus colonization (Table 2) for a 2-fold increase in effort, in contrast to typing a fixed number of colonies in all isolates (22, 23) or resampling-based methods (29). It was also able to *spa* type 100% of samples, even those previously designated nontypeable. However, ∼30% of samples with some evidence of mixed colonies had identical *spa* types in sequencing of multiple subcolonies, demonstrating the difficulty in ruling out a low proportion of samples carrying a low-frequency (e.g., 5%) cocolonizing variant. This is partly because capillary sequencing is not designed to quantify the levels of minor sequence contaminants and is relatively robust to background signal and partly because of the necessarily limited resources; even typing 24 colonies per sample would identify 5% variants only half the time (see Fig. S2 in the supplemental material). Conversely, there is little evidence for large numbers of individuals with low- to moderate-frequency (20 to 30%) cocolonization.

Cocolonization, at least with minor-frequency variants, appears to be more common than previously suggested (22). It therefore seems likely that the ability of a single strain to monopolize its host through competitive exclusion underlies the modest rates of strain replacement, as previously proposed (23), rather than this reflecting lack of transmission *per se*. The fact that individuals tended to reselect their endogenous strain in artificial decolonization/colonization studies (30) also highlights the importance of host factors, such as HLA group, which were not measured in our study.

One limitation of our study was that, due to its size, we used *spa* typing to characterize isolates, rather than more discriminatory (but expensive) whole-genome sequencing (31, 32). *spa* typing has been more widely used to date, but to our knowledge, only one study has estimated differences between *spa* types at the whole-genome level, finding that isolates ≤1 BURP distance apart could have similarly low levels of variation across the whole genome as observed within a *spa* type (32). There may also be considerable diversity within a *spa* type at the whole-genome level, which would have been missed within our study, such that individuals with one *spa* type may have more within-host diversity than others with multiple but highly related *spa* types. Although it is currently unclear whether our protocol could provide interpretable results from whole-genome sequencing, similar experiments in Clostridium difficile found that multiple sequence types could be retrieved from whole-genome sequencing of sweeps, and clones within a sequence type could also be identified if a previous database of whole-genome sequences existed (33). Of note, the *spa* types observed within this study were extremely diverse and represented most of the global lineages (24), suggesting that our results should be broadly generalizable. Further, while the number of MRSA strains was very small (reflecting the limited impact of community-associated MRSA in the United Kingdom), their patterns of colonization (Table 1) reflected those of the majority of sensitive strains (Table 2). This also supports the generalizability of our findings to nasal community carriage in general, although acquisition and carriage dynamics may differ within hospital settings. An important limitation of our study is that only nasal swabs were included, which likely missed some carriage at other sites (34, 35). However, this enabled participant self-swabbing, which was vital for our study and was recently shown to have reasonable accuracy (36).

The fact that mixed nasal colonization occurred in similar proportions of those recruitment-negative and recruitment-positive for S. aureus was not expected, but plausibly, this similar point prevalence reflects a combination of consistent exposure to diverse strains from exogenous sources (e.g., other individuals, pets, and the environment [37]) and within-host evolution. The latter, perhaps unsurprisingly, played a more important role in mixed colonization over time, with a significantly greater proportion of recruitment-positives with multiple *spa* types observed during the study having only highly related *spa* types. Nevertheless, strain replacement occurred with both highly related and unrelated strains, as exemplified by patients D and E in Table 1. Whether low-frequency variants are important sources of ongoing S. aureus transmission is unclear; if transmission potential is related to number of organisms, then low-frequency variants might also be transmitted rarely (33). The impact of mixed-strain colonization on development of clinical disease is also unknown. Since recent acquisition of a new strain versus no carriage was associated with a higher incidence of nosocomial S. aureus bacteremia, a new unrelated colonizing strain could potentially also increase the risk of invasive disease (38). Alternatively, ongoing within-host evolution could provide the opportunity for *de novo* mutations to arise and lead to clinical infection, e.g., loss-of-function mutations (39). Given the importance of invasive staphylococcal infection, both possibilities should be explored in future studies.

## Supplementary Material

Supplemental material
