# Supplementary material for: Multiple-Strain Colonization in Nasal Carriers of Staphylococcus aureus
Source: J Clin Microbiol. 2014 Apr;52(4):1192–200. doi: 10.1128/JCM.03254-13 (PMC3993518; doi:10.1128/JCM.03254-13)
Supplement: Supplemental material [file supp_52_4_1192__index.html]

Multiple-Strain Colonization in Nasal Carriers of Staphylococcus aureus — Supplemental material 

# Multiple-Strain Colonization in Nasal Carriers of Staphylococcus aureus

## Supplemental material

**Files in this Data Supplement:**

- Supplemental file 1 -

  Fig. S1 (Example traces from *spa*-typing protocol step 1), S2 (Probability of detecting mixed-strain colonization), S3 (Observed frequencies of strains from multiple colonies successfully sequenced under protocol step 3), and S4 (Longitudinal assessment of cocolonization in selected individuals) and Tables S1 (Mixed colonization with related strains versus unrelated strains versus related strains plus unrelated strains at individual time points or within individuals over the entire study)

  PDF, 411K
