# Supplementary material for: Multiple-Strain Colonization in Nasal Carriers of Staphylococcus aureus
Source: J Clin Microbiol. 2014 Apr;52(4):1192–200. doi: 10.1128/JCM.03254-13 (PMC3993518; doi:10.1128/JCM.03254-13)
Supplement: Supplemental material [file JCM.03254-13_zjm999093286so1.pdf]

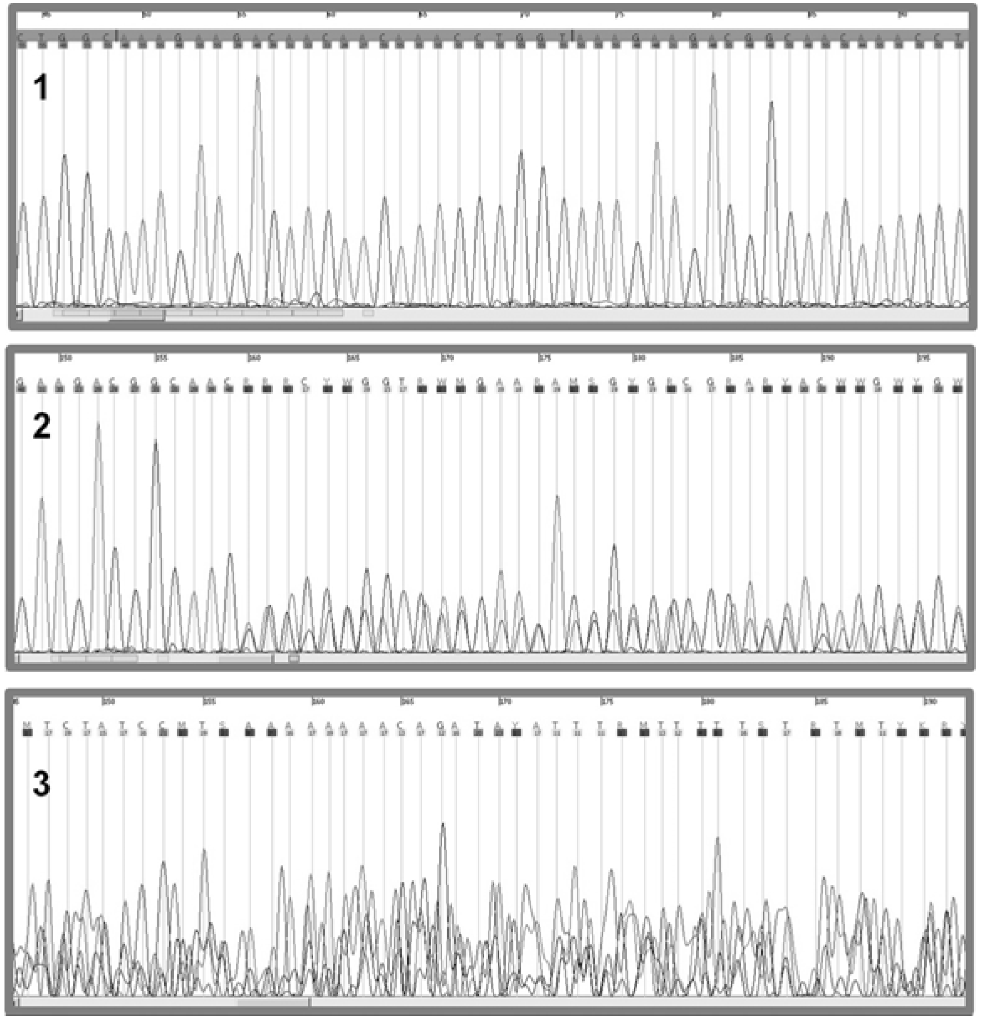

Supplementary Figure 1. Example traces from the spa-typing protocol step (1).

Note: 1. Clean sequence traces; 2. Mixed sequence traces; 3. Unreadable sequence traces.

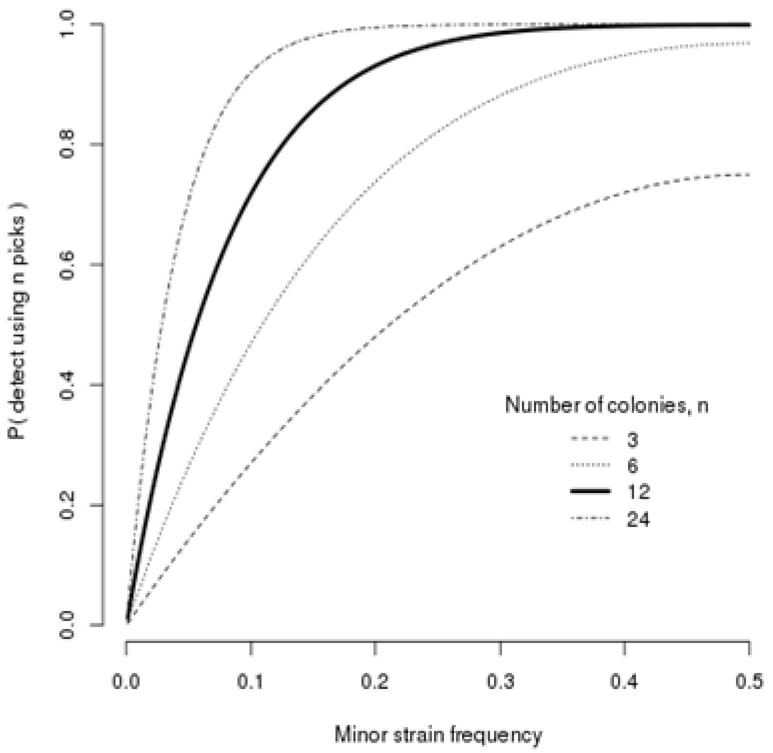

Supplementary Figure 2. Probability of detecting mixed strain colonization.

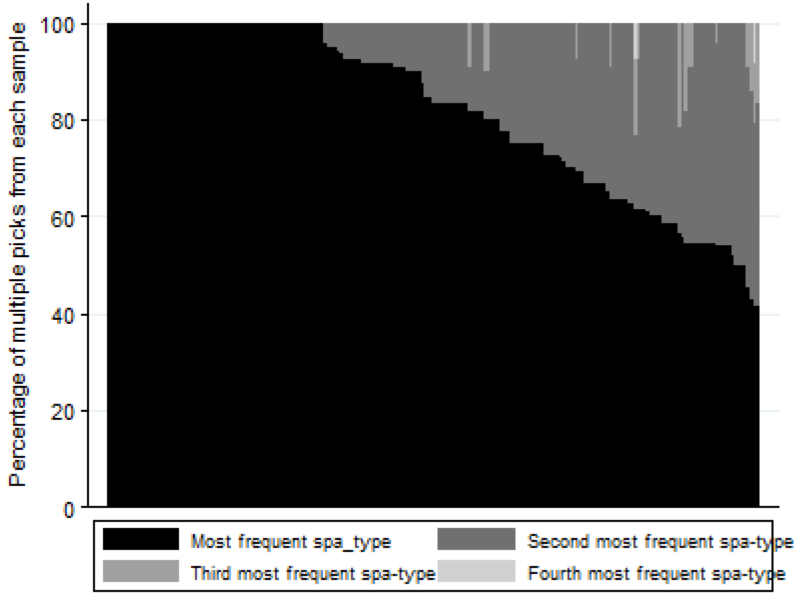

Supplementary Figure 3. Observed strain frequency from multiple colonies successfully sequenced under protocol step (3).

Note: Each sample in which multiple picks were spa-typed (n=208) is represented as a vertical line, (reverse) ordered by the proportion of the multiple picks that were the most commonly observed (dominant) spa-type. Some single-colony isolates did not produce an unambiguous spa-type so denominator for each sample is not always 12.

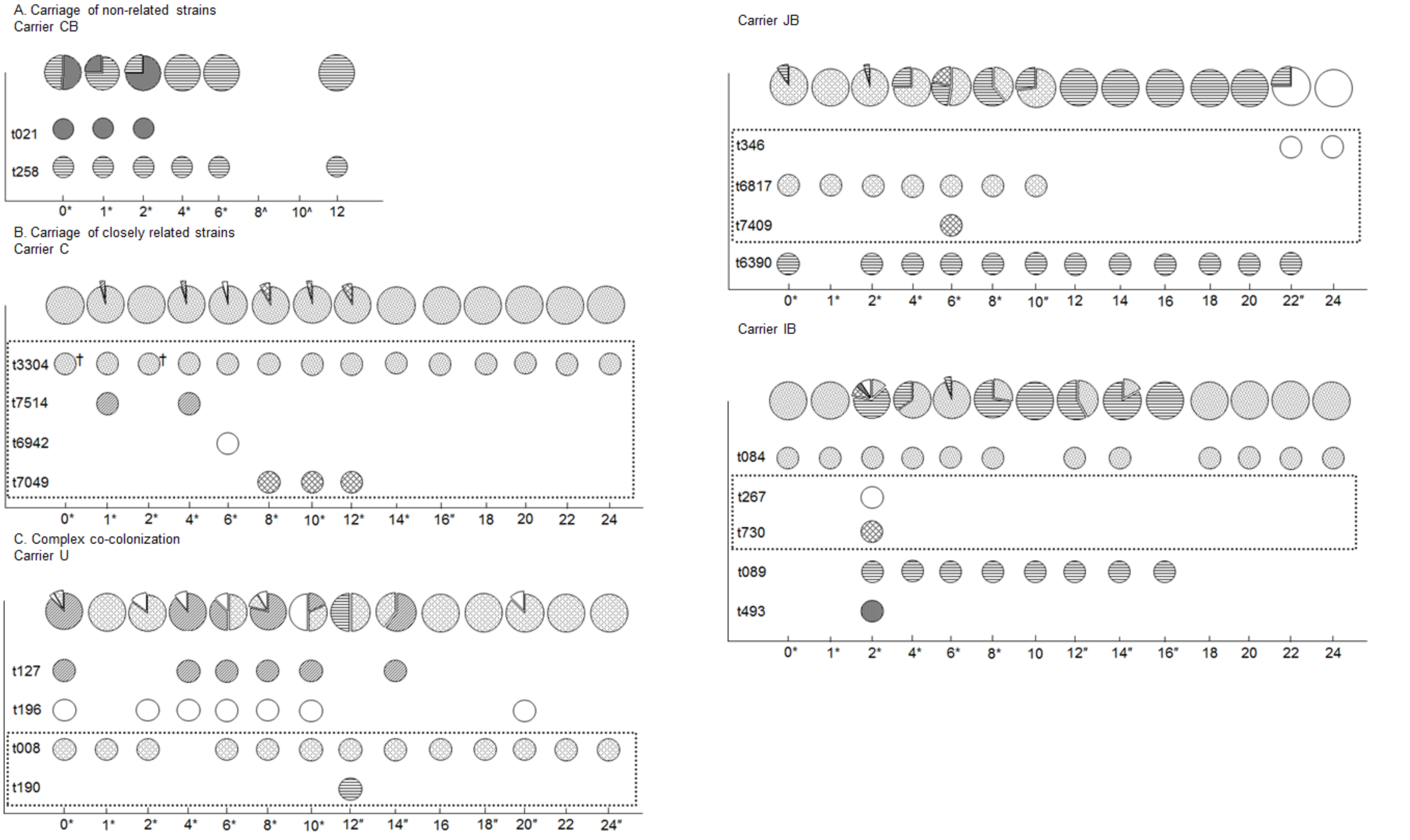

Supplementary Figure 4. Longitudinal assessment of co-colonization in selected individuals.

Supplementary Table 1. Mixed colonization with related vs. unrelated vs. related+unrelated strains at individual timepoints, or within individuals over the entire study

(a) Defining relatedness as BURP distance=6

	At individual timepoints where mixed colonization observed		For each individual over the entire study	
	Recruitment-positives	Recruitment-negatives	Recruitment-positives	Recruitment-negatives
Total*	151 (100%)	10 (100%)	123 (100%)	29 (100%)
Related only	62 (41%)	0	34 (28%)	3 (10%)
Unrelated & related	5 (3%)	0	21 (17%)	2 (7%)
Unrelated only	84 (56%)	10 (100%)	68 (55%)	24 (83%)
Comparison	Exact p=0.02		Exact p=0.03	

(b) Defining relatedness as BURP distance=4

	At individual timepoints where mixed colonization observed		For each individual over the entire study	
	Recruitment-positives	Recruitment-negatives	Recruitment-positives	Recruitment-negatives
Total*	151 (100%)	10 (100%)	123 (100%)	29 (100%)
Related only	50 (33%)	0	29 (24%)	1 (3%)

Unrelated & related	6 (4%)	0	14 (11%)	2 (7%)
Unrelated only	95 (63%)	10 (100%)	80 (65%)	26 (90%)
Comparison	Exact p=0.07		Exact p=0.02	

(c) Defining relatedness as BURP distance=2

	At individual timepoints where mixed colonization observed		For each individual over the entire study	
	Recruitment-positives	Recruitment-negatives	Recruitment-positives	Recruitment-negatives
Total*	151 (100%)	10 (100%)	123 (100%)	29 (100%)
Related only	30 (20%)	0	19 (15%)	0
Unrelated & related	4 (3%)	0	11 (9%)	2 (7%)
Unrelated only	117 (77%)	10 (100%)	93 (76%)	27 (93%)
Comparison	Exact p=0.39		Exact p=0.05	

(d) Defining relatedness as BURP distance=1

	At individual timepoints where mixed colonization observed		For each individual over the entire study	
	Recruitment-positives	Recruitment-negatives	Recruitment-positives	Recruitment-negatives
Total*	151 (100%)	10 (100%)	123 (100%)	29 (100%)

Related only	27 (18%)	0	14 (11%)	0
Unrelated & related	5 (3%)	0	12 (10%)	2 (7%)
Unrelated only	119 (79%)	10 (100%)	97 (79%)	27 (93%)
Comparison	Exact p=0.43		Exact p=0.14	

* Total refers to the number of timepoints where >1 spa-type was observed in an individual for the first 2 columns, and to the total number of individuals with >1 spa-types isolated during the 24 months study (at the same or different timepoints) for the last two columns. Individuals with multiple spa-types over the study could have had the same or different strains isolated at individual timepoints, or only single spa-types at each timepoint but multiple spa-types over the study.
